# Does the priority of ambulance guarantee no delay? a MIPSSTW model of emergency vehicle routing optimization considering complex traffic conditions for highway incidents

**DOI:** 10.1371/journal.pone.0301637

**Published:** 2024-04-18

**Authors:** Siliang Luan, Zhongtai Jiang

**Affiliations:** 1 School of Civil Engineering, Qingdao University of Technology, Qingdao, Shandong, China; 2 Engineering Research Center of Concrete Technology Under Marine Environment, Ministry of Education, Qingdao, Shandong, China; 3 School of Transportation, Jilin University, Changchun, Jilin, China; Southwest Jiaotong University, CHINA

## Abstract

Globally, traffic accidents on the highway network contribute significantly to a high fatality rate, drawing considerable attention from health institutions. The efficiency of transportation plays a vital role in mitigating the severe consequences of these incidents. This study delves into the issues of emergency vehicles experiencing delays despite having priority. Therefore, we construct mixed-integer linear programming with semi-soft time windows (MIPSSTW) model for optimizing emergency vehicle routing in highway incidents. We analyze the time-varying and complex traffic situations and respectively propose corresponding estimation approaches for the travel time of road segments, intersections on the urban road network, and ramp-weave sections on the highway network. Furthermore, we developed a modified cuckoo search(MCS) algorithm to solve this combinatorial problem. Optimization strategies of Lévy flight and dynamic inertial weight strategy are introduced to strengthen the exploration capability and the diversity of solution space of the CS algorithm. Computational experiments based on the Chinese emergency medical system data are designed to validate the efficacy and effectiveness of the MIPSSTW model and MCS algorithm. The results show that our works succeed in searching for high-quality solutions for emergency vehicle routing problems and enhance the efficacy of strategic decision-making processes in the realm of incident management and emergency response systems.

## Introduction

The World Health Organization estimated that approximately 1.3 million people lose their lives each year as a result of road traffic crashes [1]. The distribution of traffic injuries is not uniform globally. Almost 90% of traffic-related fatalities take place in countries with lower and middle-income levels [2]. Incidents on the highway networks have been rising at an alarming rate, posing a growing threat to public safety, and requiring urgent attention from transportation authorities. The surge in accidents, ranging from collisions and breakdowns to hazardous material spills, has the characteristics of suddenness, unexpectedness, and serious socio-economic damage. Globally, traffic accidents on the highway network cause major fatality rates and are of concern to health institutions. Addressing the aftermath of such incidents, emergency medical services (EMS) systems play a vital role in public health emergencies (PHEs) intending to minimize serious impacts on affected individuals. The seamless coordination and efficiency of EMS operations are crucial in providing timely and effective medical assistance to those involved in traffic accidents, contributing to overall public health and safety. Effectively solving the emergency vehicle routing problems enhances the ability of the EMS system to navigate through complex road networks, reaching accident sites promptly and providing life-saving interventions. To this effect, the early works related to emergency rescue problems focus on the emergency vehicle dispatch problem of prehospital emergency care. It is a common belief that emergency vehicles have traveling priority when their lights and sirens are activated. Other road users are generally expected to yield the right of way and make way for these emergency vehicles to pass quickly and safely. However, the timely movement of emergency vehicles might be subject to delays caused by saturated or oversaturated traffic conditions. Meanwhile, there is a scarcity of literature addressing the investigation of rescue routes for highway accidents. Consequently, the strategic optimization of emergency vehicle routing for highway incidents advances the overarching objective of public health and safety.

This paper seeks to provide some empirical evidence from China to the existing literature by conducting mixed-integer linear programming with semi-soft time windows (MIPSSTW) model for emergency vehicle routing optimization in the rescue process for highway incidents. The study explores the travel time from the ambulance base to a highway incident scene, with an emphasis on the disruptions posed by varying traffic conditions. We use the improved Bureau of Public Roads (BPR) model for the estimation of travel time of road segments, the queuing theory for the evaluation of travel time for intersections on the urban road network, and the improved fixed detector method for ramp-weave sections on the highway network. In addition to strengthening the role of models in routing research, the focus on the utilization of the modified Cuckoo Search (MCS) algorithm. This nature-inspired metaheuristics algorithm, rooted in natural phenomena and biological behaviors, is a good tool to solve this non-deterministic polynomial (NP) problem. The MCS algorithm incorporates an advanced selection strategy based on neighborhood structures and a dynamic inertial weight strategy to maintain the diversity of the solution space. The results of this study may shed light on future emergency vehicle routing optimization in decision support systems.

The rest of this paper is structured as follows. Section 2 gives a literature review. Section 3 presents our MIPSSTW model considering the complex traffic conditions. In section 4, an MCS algorithm is presented to solve this combinatorial problem. A case study in China and the results of computational experiments are described and analyzed in section 5. In section 6, this paper gives concluding remarks and recommendations for future works.

## Related literature

The basic description of VRP involves vehicles departing from a distribution center, delivering goods to customers or delivery stations in a pre-planned sequence, and then returning to the distribution center. It is a very costly procedure constrained by service windows and achieving single or multiple objectives, such as minimizing total time, costs, and so on. The early study of VRP was proposed by Dantzig and Ramser in 1959 concerning the optimum routing for gasoline delivery trucks [3]. The shortest path between the bulk terminal and the service station is solved by the linear programming formulation. The VRP problem was later extensively researched in various fields such as management science [4,5], operation research [6], logistics science [7,8], and computer science [9,10]. Since then, a significant number of variations have been explored, including hard [11,12], soft [13], and fuzzy service time windows [14]. Hard time windows require that the delivery task needs to be finished during the designated time frame of the time windows, or else an infeasible solution will be obtained. Soft time windows, on the other hand, allow that the delivery task is not able to be completed within a certain time range, but it can be violated at a certain penalty [15]. Fuzzy time windows refer to the scheduling procedure in which tasks are not precisely defined but rather have a degree of flexibility. The hard, soft and fuzzy time windows may not be suitable during emergencies, since incidents often request immediate responses and allow little flexibility in terms of rescue time. Meanwhile, emergency planning needs to be clear, simple, and straightforward for faster decision-making. Soft and fuzzy time windows introduce uncertainty, potentially increasing the complexity of planning and making execution more challenging. Additionally, emergencies are often characterized by their dynamic and unpredictable nature and situations. However, the rigid constraints of hard time windows are not able to adapt to the quickly changing circumstances and urgent requirements. Therefore, it is important to develop an advanced model with approaciate constrains for the vehicle routing problems with time windows(VRPTW) problem, especially applying in emergency vehicle routing optimizations.

There is a large literature using known and fixed costs or travel time without considering other crucial aspects like traffic conditions in emergency VPR [16]. Jin et al. proposed a system that incrementally generates the optimal path for rescue vehicles. It utilizes a Temporal Convolutional Network (TCN) to calculate time-varying ratios between toll booths and road segments and employs a dynamic BPR function along with the Dijkstra algorithm to generate real-time optimal routes [17]. Yoon and Albert formulated a Markov decision process model for the EMS system aiming to rapidly respond to patients and deploy appropriate personnel to patients based on their healthcare needs [18]. The objective primarily aims to minimize total distance, which is equated with minimizing travel time by assuming constant vehicle speed. Nevertheless, vehicle speed fluctuates over time, and traffic can be unpredictable. Even though emergency vehicles have priority right of way, the unpredictability of traffic congestion might have an impact on rescue delays. Musolino et al. designed a framework of two modeling components for emergency vehicles considering within-day variations of link travel times [19]. Oza and Tech proposed a strategy of emergency response vehicle preemption to facilitate the timely and non-disruptive response of emergency vehicles. The strategy could leverage connected infrastructure and manage traffic before, during, and after emergency vehicle traversal to minimize traffic delays [20]. Tan et al. developed an emergency logistics vehicle routing model for the novel coronavirus pandemic and minimized travel costs, time costs, early/late punishment costs, and fixed costs in their model [21]. Min et al. focused on the medical emergency supplies dispatching considering the demand urgency and designed an improved cuckoo-ant colony hybrid algorithm to solve the problem [22]. However, these studies rarely consider that the saturated traffic conditions might pose a challenge to emergency decision-makers, since the queuing phenomena on the vehicle merging and diverging areas cause the rescue delay. In addition, current researches rarely specifically discuss the rescue process for highway incidents. Thus, the characteristics of traffic flow on ramp-weave sections and their serious impact on emergency rescue delay, which might not be given enough attention.

A large number of papers develop algorithms for VRP problems. The typical classic algorithms include Dijkstra, A*, Floyd, and approaches based on graph theory. In addition, there are numerous heuristic algorithms for solving complex models, such as genetic algorithms(GA), ant colony (AC) algorithms, particle swarm (PS) algorithms, and so on. Subsequently, scholars have consistently enhanced and evolved classical algorithms. However, the difficulties in the solutions to large-scale problems still persist. These algorithms might fall into local optima, low operational efficiency, and weak robustness. Addressing these challenges requires continued research for enhancements or innovative approaches in the optimization of emergency vehicle paths. CS algorithm is a metaheuristic swarm-based approach proposed by Yang and Deb to emulate the interesting feature of parasitism of cuckoo species [23]. The CS algorithm has several advantages over other algorithms. Firstly, it requires fewer initial search configuration parameters, so it is relatively simple to implement. Secondly, the CS algorithm incorporates Lévy flights for random walks, which effectively balances exploration and exploitation for global solutions. Lastly, the CS algorithm is an efficient metaheuristic algorithm that can be adapted and extended for various optimization scenarios by modifying the related parameters [24]. However, the standard CS algorithm has a large gap in slow convergence speed and search abilities. Thus, CS-based variant algorithms have been developed to get satisfactory solutions from different perspectives. Ouaarab et al. extended a discrete CS algorithm to strengthen the intensive search for the best solutions of the population [25]. Xiao et al. proposed an improved CS algorithm by two heuristics for the quality of the initial population to optimize the routes of patient transportation [26]. Li et al. developed a novel CS extension with Q-learning step size and genetic operator and demonstrated their algorithm is a competitive swarm algorithm on effectiveness [27]. Yang et al. proposed a CS variant with a modified operation mode(CCSMO) to construct a collaborative mechanism to enhance the information sharing between cuckoos [28]. From the above surveys, few papers have been discussed on VRP with semi-soft constraints and considering complex traffic conditions. Therefore, it is worthwhile to develop a CS-based algorithm to solve this research problem and improve the quality of solutions and the convergence speed of the standard CS algorithm.

## Problem description and model formulation

This section includes the problem description, assumptions, the notation used in the model, and our mathematical model.

### 3.1 Problem description

In this study, the VRPTW is framed as a critical routing challenge in the rescue process for highway incidents. The objective of the problem is to find optimal routes in terms of the response time. As previously mentioned, the constraints of hard and soft time windows are usually set for routing optimization. However, these constraints may face limitations for the practical application. The daily scheduling of EMS systems is faced with mass incidents of unknown causes and other sudden uncertainties. The ambulances might be occupied and unavailable for immediate emergency response during the peak hour. Therefore, the susceptibility of infeasible time window constraints to violation makes the constraints of hard time windows unsuitable for an EMS operation system. Motivated by the characteristics of the constraints of soft time windows, we design a programming model with the constraints of semi-soft time windows for emergency vehicle path optimization. Soft time windows provide a higher degree of flexibility without imposing penalties or negative consequences for deviations. Semi-soft time windows we used in this paper strike a balance between flexibility and adherence to certain constraints. The rescue time threshold of the prehospital emergency healthcare system restricts the pernicious effects caused by excessive deviation, so semi-soft time windows could satisfy the requirements of emergency management and it still allows flexibility for exceptional scenarios. In our paper, the model featuring semi-soft time window constraints incorporates distinct response time threshold values tailored to different priority levels of patients or events. Penalties are incurred when exceed specific time window frames, which ensures a practical and adaptive model for emergency vehicle path optimization.

Incidents occurring on highway networks significantly impact mortality and morbidity rates, primarily due to the high speeds involved [29]. Hospitals and ambulance bases are typically situated in urban or rural areas rather than within service zones along highway networks. The ambulance service operates on a comprehensive model, requiring departures from rescue bases or the site of the latest incident, navigating to the current incident location, and ultimately reaching the designated hospitals. In this intricate process, ambulance routes traverse both urban roads and highways. Consequently, it becomes imperative to consider the traffic conditions in both urban and highway environments when responding to incidents on the highway network.

During non-peak hours, ambulances and other emergency vehicles generally encounter minimal obstruction, as drivers are obligated to yield the right of way to these vehicles. However, exceptions occur during peak hours or particular holiday travel periods when heavy traffic congestion significantly contributes to delays for emergency vehicles on the road networks. Yielding to emergency vehicles may become challenging or even impossible, since it’s difficult for drivers to pull over or exit the road promptly. As a result, shoulders or emergency lanes are strategically designed to facilitate the passage of emergency vehicles. These lanes allow emergency vehicles to navigate around traffic congestion on busy multi-lane roads within highway networks, which ensures a smoother and more efficient response during peak hours.

Despite emergency vehicles having the right of way in the road network and the capability to automatically trigger changes in traffic light sequences, they must exercise caution in selecting routes to minimize potential delays. The emergency vehicles try to avoid road section with poor traffic reliability and high traffic impedance such as the areas near heavily congested schools and construction zones. Opting for paths that allow them to navigate around traffic congestion enhances the efficiency of emergency rescue efforts. However, the routing of emergency vehicles is inevitably susceptible to losses caused by unexpected traffic jams, particularly at two critical nodes: intersections within urban road networks and entrance/exit ramps on the highway networks. In instances where conditions at intersections are saturated or oversaturated, the resulting significant impacts on network performance—such as delays, queues, and reduced capacity—can impede the overall efficiency of emergency rescue operations [30]. Meanwhile, when ambulances enter or exit ramps, they must adjust their speed in accordance with the flow of upstream and downstream traffic around the ramp-weave sections. This careful consideration and strategic navigation are crucial for ensuring the smooth progress of emergency vehicles through these points on the road.

Our study is conducted on a complete directed graph *G* = (*N*, *E*) containing a set of nodes *N* and a set of arcs *E*. We develop an MIPSSTW model with the constraints of the semi-soft time windows considering the travel time of both nodes and arcs. The nodes refer to the intersections on the urban road networks and entrance/exit ramps on the highway networks, and arcs correspond to road sections within the study areas. The approach to the prediction of travel time and queueing time for the intersections is based on queuing theory. We employ an improved fixed detector method to estimate the travel time of emergency vehicles passing through ramp-weave sections. The improved Bureau of Public Roads (BPR) model serves as a valuable tool for predicting the travel time of road sections. This comprehensive approach enhances the precision and reliability of our study in modeling and optimizing emergency vehicle routes.

Assumptions and symbolic descriptions

Throughout this paper, the following assumptions are consistently applied:

1. The ambulance, origin, and destination are designated by the EMS system in the call center (or dispatch center), and thus, these pieces of information are considered known parameters.
2. If the paramedics in the ambulance can carry out the on-site treatment when the ambulance arrives at the accident scene, the rescue will be finished. If further treatment is required based on the severity of the patient, the ambulance will transport the patient to the hospital.
3. The urgency priority of the accident can be determined by the dispatch center.
4. The routing problem doesn’t consider the emergency call queue time, the paramedics’ preparation time, patient boarding and alighting time during the response process.
5. The travel time of emergency vehicles is also associated with the driver’s driving habits and personality. In our study, we assume each ambulance driver’s driving habits are consistent.
6. We assume that each ambulance is dedicated to serving only one incident.
7. This model doesn’t consider that the road network is disrupted due to natural disasters, such as hurricanes, earthquakes, tsunamis, etc.

Based on the above assumptions, we propose the MIPSSTW model. Table 1 summarizes all the notations we used in the MIPSSTW model.

**Table 1:** Notation used in the model.

Index sets	Description
*N*	Set of notes on the road network, N={0,1,2,…,n,n+1}
*E*	Set of road segments(arcs) that connect between two notes *N*, *E*⊆*N*×*N*
*G*	Directed graph of the road network, *G* = (*N*, *E*)
Parameters	
*c*	The number of lanes at intersections.
*ω*_1_,*ω*_2_	The estimated parameters by LSTM neural network.
*α*,*β*	Dimensionless parameters.
$\varphi_{l}^{k}$	The rescue ability of vehicle *k* for the response priority *l*.
*d*_*a*_	The demand on the accident *a*.
*ψ*	The weight of delay penalty for the response priority *l*.
*m*_*ij*_	The maximum number of vehicles that can pass through the intersection from node *i* to node *j*.
*λ*_*ij*_	The rate of vehicle arrivals at the entry of the road section (*i*,*j*).
*μ*_*ij*_	The maximum traffic capacity per unit time at the intersection downstream of road segment (*i*,*j*).
*g*_*ij*_	The green signal ratio in the direction of travel at the intersection downstream of road segment (*i*,*j*).
*η*	The number of queuing vehicles.
*ρ*_*ij*_(*t*)	The traffic service level of road segment (*i*,*j*) at time *t*.
*L*_*ij*_(*t*)	The queue length of road segment (*i*,*j*) at time *t*.
$\bar{s_{i}}$	The closest upstream detector location of the highway ramp.
*s* _ *i* _	The closest downstream detector location of the highway ramp.
$v_{\bar{s_{i}}}(t)$	The vehicle speed detected by the upstream detector location $\bar{s_{i}}$ at time *t*.
$v_{\underline{s_{i}}}(t)$	The vehicle speed detected by the downstream detector location *s*_*i*_ at time *t*.
$\bar{D_{i}}$	The length of road segment from the upstream detector location $\bar{s_{i}}$ to the intersection point of highway segment and ramp.
*D* _ *i* _	The length of road segment from the intersection point of highway segment and ramp to the downstream detector location *s*_*i*_.
*Z*(*ξ*)	The objective function of path optimization under the scenario *ξ*.
$x_{ij}^{k}$	Equal to 1 if vehicle *k* traverses are (*i*,*j*), and 0 otherwise.
*τ*_*ij*_	The travel time from node *i* to node *j*.
*f*_*k*_	The penalty of delay of vehicle *k*.
*θ*_*ij*_	The node travel time from node *i* to node *j*.
$\theta_{ij}^{1}$	The queuing travel time between node *i* and node *j*.
$\theta_{ij}^{2}$	The intersection travel time between node *i* and node *j*.
*T*_*ij*_	The travel time of road section between node *i* and node *j*.
T	The actual travel of ordinary vehicles.
$T_{f}$	The travel time of free flow.
*q*	The current traffic volume.
*Q*	The improved expression of current traffic volume.

### 3.2 Mathematical model

Based on the aforementioned assumptions and notation, we formulate a MIPSSTW model to optimize the routing problem of ambulances for highway incidents, outlined as follows:

$$Z(\xi )=\min\sum_{k\in K}\left(\sum_{i\in N}\sum_{j\in N}\tau_{ij}x_{ij}^{k}+f_{k}\right)$$ (1)

$$st.\;\sum_{k\in K}x_{ij}^{k}=1\;\forall i,j\in N,\forall k\in K$$ (2)

$$\sum_{j\in N}x_{ij}^{k}=\sum_{j\in N}x_{ji}^{k}\;\forall k\in K$$ (3)

$$\sum_{k\in K}\sum_{j\in N}\varphi_{l}^{k}x_{ja}^{k}\geq d_{a}\;\forall k\in K$$ (4)

$$f_{k}=\{\begin{array}{c} \psi_{1}(\sum_{i\in N}\sum_{j\in N}t_{ij}-\Delta t_{high})\;\sum_{i\in N}\sum_{j\in N}t_{ij}\geq \Delta t_{high}\\ \psi_{2}(\sum_{i\in N}\sum_{j\in N}t_{ij}-\Delta t_{low})\;\sum_{i\in N}\sum_{j\in N}t_{ij}\leq \Delta t_{low} \end{array}$$ (5)

$$x_{ij}^{k}\in \{0,1\}\;\forall i,j\in N,i\neq j,\forall k\in K$$ (6)

The objective function (1) aims to minimize the total travel time and delay time of the rescue routing. Constraints (2) ensure that every incident receives a response from emergency vehicles. Constraints (3) guarantee the traffic flow balance for intermediate nodes. Constraints (4) ensure that emergency vehicles with different rescue abilities can meet the needs of each incident. Function (5) represents the constraints of semi-soft time windows. Where *ψ*_1_,*ψ*_2_ respectively denotes the weight of delay penalties for high- and low-priority requests. More precisely, we establish various time thresholds for the incident requests with different priorities. The semi-soft time windows penalize delays based on the variance between the actual travel time and the time threshold. This approach allows for a comprehensive consideration of the adverse impact of delays on events and patient conditions at different levels. Constraints (6) impose the attributes of binary decision variables.

It noted that *τ*_*ij*_ denotes the total travel time spent on nodes *i*,*j* and arcs (*i*,*j*). Therefore, it can be expressed as:

$$\tau_{ij}=\theta_{ij}+T_{ij}$$ (7)

Where *θ*_*ij*_ denotes the node time consumption from node *i* to node *j*, and *T*_*ij*_ represents the travel time of road segment (*i*,*j*). We intend to use the improved BPR model to evaluate the travel time of road segments.

The classic BPR model was established by the U.S. Federal Highway Administration through regression analysis of highway historical highway data [31]. Due to its straightforward formulation for traffic impedance, this model is widely applied in transportation research. The expression of the classic BPR model is as follows:

$$T=T_{f}[1+\alpha {\left(\frac{q}{C}\right)}^{\beta }]$$ (8)

As the classic BPR model is developed from the highway traffic flow data, it is not suitable for saturated and oversaturated traffic conditions. In addition, the trend of vehicle speed typically shows an initial increase followed by a decrease with the increase of traffic flow. Obviously, the monotonic speed variations in the model doesn’t accurately reflect reality. The reason for this phenomenon is that the actual traffic flow is significantly higher than the design flow. The discrepancy arises because the actual traffic flow surpasses the design flow. Therefore, it is necessary to improve the classic BPR model to better suit real-life situations. The travel time of the road segment is expressed in Formula (9).

$$T=T_{f}[1+\alpha {\left(\frac{Q}{C}\right)}^{\beta }]$$ (9)

Where *Q* is the modified value of traffic volume:

$$Q'=\{\begin{array}{l} q\;q\leq C\\ 2C-q\;q>C \end{array}$$ (10)

Furthermore, *α* and *β* are also recalculated by new approaches as follows:

$$\beta =\pm \frac{\omega_{1}(Q/C)}{\omega_{2}+(Q/C)}$$ (11)

$$\alpha =(T/T_{f})-1$$ (12)

Where *ω*_1_,*ω*_2_ are the estimated parameters by the LSTM neural network [32,33].

In our paper, we emphasize the importance of node time consumption including intersections on the urban road networks and ramp-weave sections on the highway networks. We propose two distinct approaches to discuss these conditions separately.

1. When the node is an intersection on the urban road network

Despite emergency vehicles having priority of the way, delays may occur due to signal timing at intersections. When traffic volume is excessively high, the road network may reach a saturated or oversaturated status resulting in significant queuing. This might further contribute to emergency vehicle delays. In other words, the travel time of emergency vehicles is influenced by the traffic flow of the road network. Therefore, it is crucial to predict the travel time of intersections to prevent unnecessary delays. This node travel time *θ*_*ij*_ is divided into the queue waiting time $\theta_{ij}^{1}$ and the travel time $\theta_{ij}^{2}$ through the intersection:

$$\theta_{ij}=\theta_{ij}^{1}+\theta_{ij}^{2}$$ (13)

The vehicles at the intersection adhere to an $M/M/c/m_{ij}$ queuing system. The vehicle arrival rate *λ*_*ij*_(*t*) conforms to the Poisson distribution, and the traffic service rate *μ*_*ij*_(*t*) follows a negative exponential distribution. We assume that the maximum number of vehicles *m*_*ij*_ can pass through the intersection. The queue system is designed to prevent the number of new arrival vehicles from exceeding this maximum capacity *m*_*ij*_. Given the steady state of the traffic conditions, the transition probabilities of vehicles also remain steady and constant. We define this transition probability as $\lambda_{ij}(t)\cdot P_{ij}^{\left(0\right)}(t)$. Meanwhile, the queuing time for other vehicles is associated with the timing of intersection signals. *g*_*ij*_(*t*) denotes the green signal ratio of the intersection in the travel direction of road section (*i*,*j*), and the intersection’s effective service rate can be expressed as $g_{ij}(t)\mu_{ij}(t)$. $g_{ij}(t)\mu_{ij}(t)$ can also be interpreted as the transition probability of the intersection state. According to the queueing theory, the probability of *η* queuing vehicles is:

$$P_{ij}^{\eta }(t)=\{\begin{array}{c} \frac{{\left(c\rho_{ij}(t)\right)}^{\eta }}{\eta !}P_{ij}^{\left(0\right)}(t)\;0\leq \eta <c\\ \frac{c^{c}}{c!}\rho_{ij}^{\eta }(t)P_{ij}^{\left(0\right)}(t)\;c\leq \eta \leq m_{ij} \end{array}$$ (14)

When normalizing Eq (14), it can be expressed as:

$$\sum_{\eta =0}^{m_{ij}}P_{ij}^{\left(\eta \right)}(t)=1$$ (15)

$$\sum_{\eta =0}^{m_{ij}}P_{ij}^{\left(\eta \right)}(t)=P_{ij}^{\left(0\right)}(t)[\sum_{\eta =0}^{c-1}\frac{{\left(c\rho_{ij}(t)\right)}^{\eta }}{\eta !}+\sum_{\eta =c}^{m_{ij}}\frac{c^{c}}{c!}\rho_{ij}^{\eta }(t)]$$ (16)

$$P_{ij}^{\left(0\right)}(t)=\{\begin{array}{c} {\left[\sum_{\eta =0}^{c-1}\frac{{\left(c\rho_{ij}(t)\right)}^{\eta }}{\eta !}+\frac{{\left(c\rho_{ij}(t)\right)}^{c}}{c!}\cdot \frac{1-\rho_{ij}^{m_{ij}-c+1}(t)}{1-\rho_{ij}(t)}\right]}^{-1}\rho_{ij}(t)\neq 1\\ {\left[\sum_{\eta =0}^{c-1}\frac{c^{\eta }}{\eta !}+\frac{c^{c}}{c!}\cdot (m_{ij}-c+1)\right]}^{-1}\;\rho_{ij}(t)=1 \end{array}$$ (17)

Where *ρ*_*ij*_(*t*) denotes the traffic intensity. Traffic intensity is a significant metric for traffic management and planning, providing a measure of the congestion level in the road networks. The expression of traffic intensity *ρ*_*ij*_(*t*) is shown in Formula (18).


$$\rho_{ij}(t)=\frac{\lambda_{ij}(t)}{cg_{ij}(t)\mu_{ij}(t)}$$ (18)

The number of queuing vehicles *L*_*ij*_(*t*) is:

$$L_{ij}(t)=\{\begin{array}{ll} \frac{c^{c}\rho_{ij}^{c+1}(t)P_{ij}^{\left(0\right)}(t)}{c!{\left(1-\rho_{ij}(t)\right)}^{2}}[1-(m_{ij}-c+1)\rho_{ij}^{m_{ij}-c}(t)+(m_{ij}-c)\rho_{ij}^{m_{ij}-c+1}(t)] & \rho_{ij}(t)\neq 1\\ \frac{c^{c}}{2c!}(m_{ij}-c)(m_{ij}-c+1)P_{ij}^{\left(0\right)}(t) & \rho_{ij}(t)=1 \end{array}$$ (19)

Based on the Little’s law [34], the average queue waiting time $\theta_{ij}^{1}(t)$ is derived:

$$\theta_{ij}^{1}(t)=\frac{L_{ij}(t)}{\lambda_{ij}(t)(1-P_{ij}^{m_{ij}}(t))}$$ (20)

$$=\{\begin{array}{l} \frac{c^{c}\rho_{ij}^{c+1}(t)P_{ij}^{\left(0\right)}(t)}{c!{\left(1-\rho_{ij}(t)\right)}^{2}\lambda_{ij}(t)(1-P_{ij}^{m_{ij}}(t))}[1-(m_{ij}-c+1)\rho_{ij}^{m_{ij}-c}(t)+(m_{ij}-c)\rho_{ij}^{m_{ij}-c+1}(t)],\rho_{ij}(t)\neq 1\\ \frac{c^{c}P_{ij}^{\left(0\right)}(t)}{2c!\lambda_{ij}(t)(1-P_{ij}^{m_{ij}}(t))}(m_{ij}-c+1)(m_{ij}-c),\;\rho_{ij}(t)=1 \end{array}$$

The travel time for emergency vehicles to pass through the intersection $\theta_{ij}^{2}(t)$:

$$\theta_{ij}^{2}(t)={\left(g_{ij}(t)\mu_{ij}(t)\right)}^{-1}$$ (21)

1. When the node is a ramp-weave section on the highway road network.

On- and off-ramps constitute typical weaving segments, resulting from the convergence and divergence of traffic streams. Weaving congestions easily occur and generate bottlenecks in the road networks due to the transfer behavior of drivers and the turbulence of traffic flow. Therefore, the average speed of emergency vehicles differs between weaving sections and freeways. In our study, the fixed detector method is employed to estimate the travel time of ambulances on highway entrance and exit ramps. The parameters indicative of the traffic operating conditions can be obtained through fixed detectors installed on the highway network, including vehicle velocity, traffic occupancy, and more. We calculate the travel time of emergency vehicles passing through ramp-weave sections using these traffic parameters obtained from fixed detectors.

**Fig 1:**
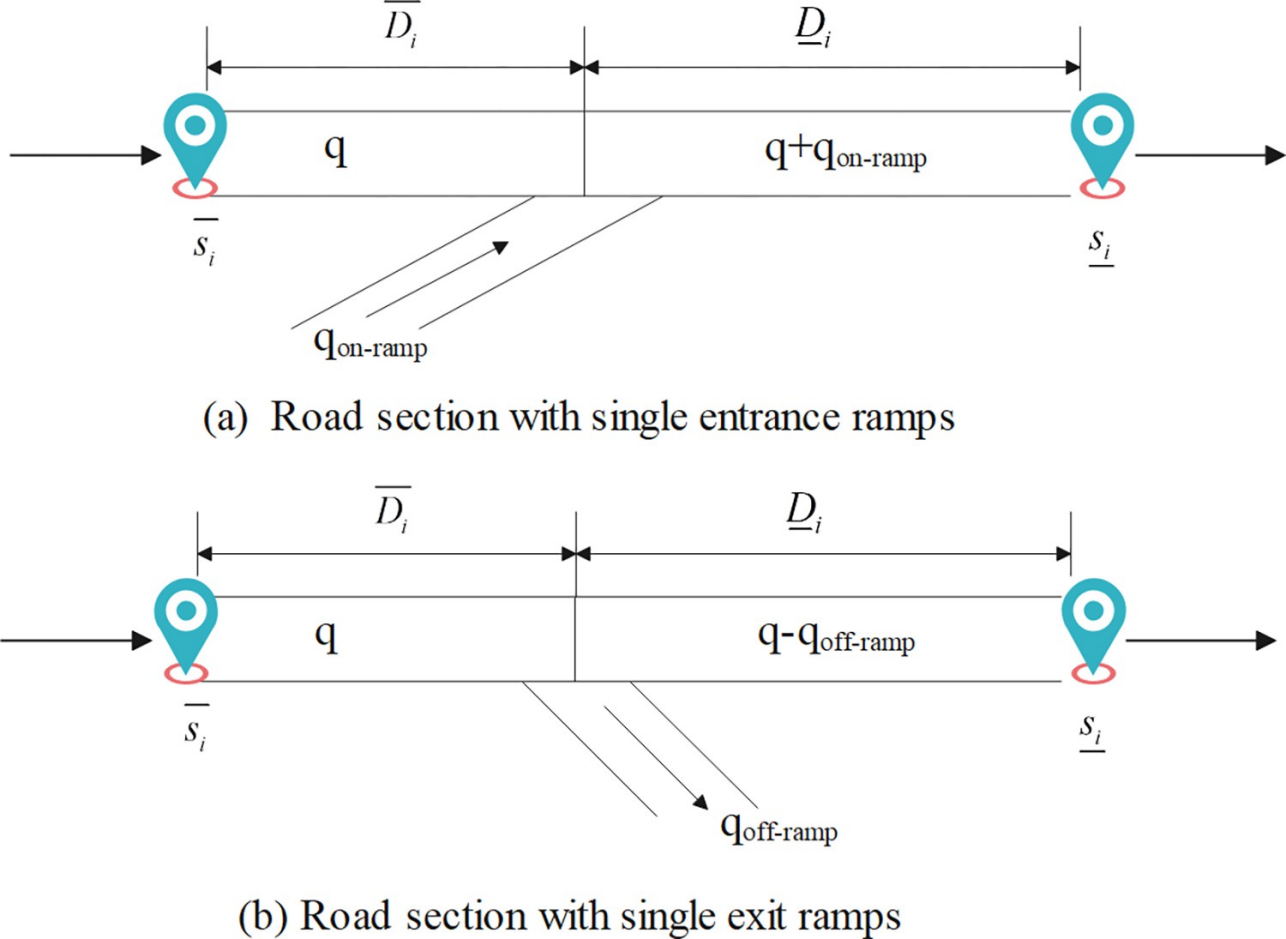
The first type of ramp-weave section.

In this study, we categorize two types of ramp-weave sections based on their geometric configuration for the placement of detectors. As shown in Fig 1, the first type of ramp-weave section includes a single entrance or exit ramp. Due to variations in traffic flow, the speed of ambulance changes only once. Given that *q*_*on−ramp*_ denotes the travel flow entering the on-ramp and *D*_*i*_ denotes the distance from the location of downstream detectors *s*_*i*_ to the junction of the entrance ramp and the highway segment. The total traffic flow on this road section can be expressed as *q*+*q*_*on−ramp*_. The travel time *Q*_*i*_(*t*) of emergency vehicles at node *i* is:

$$\theta_{i}(t)=\frac{\bar{D_{i}}}{v_{\bar{s_{i}}}(t)}+\frac{{\underline{D}}_{i}}{v_{\underline{s_{i}}}(t)}$$ (22)

**Fig 2:**
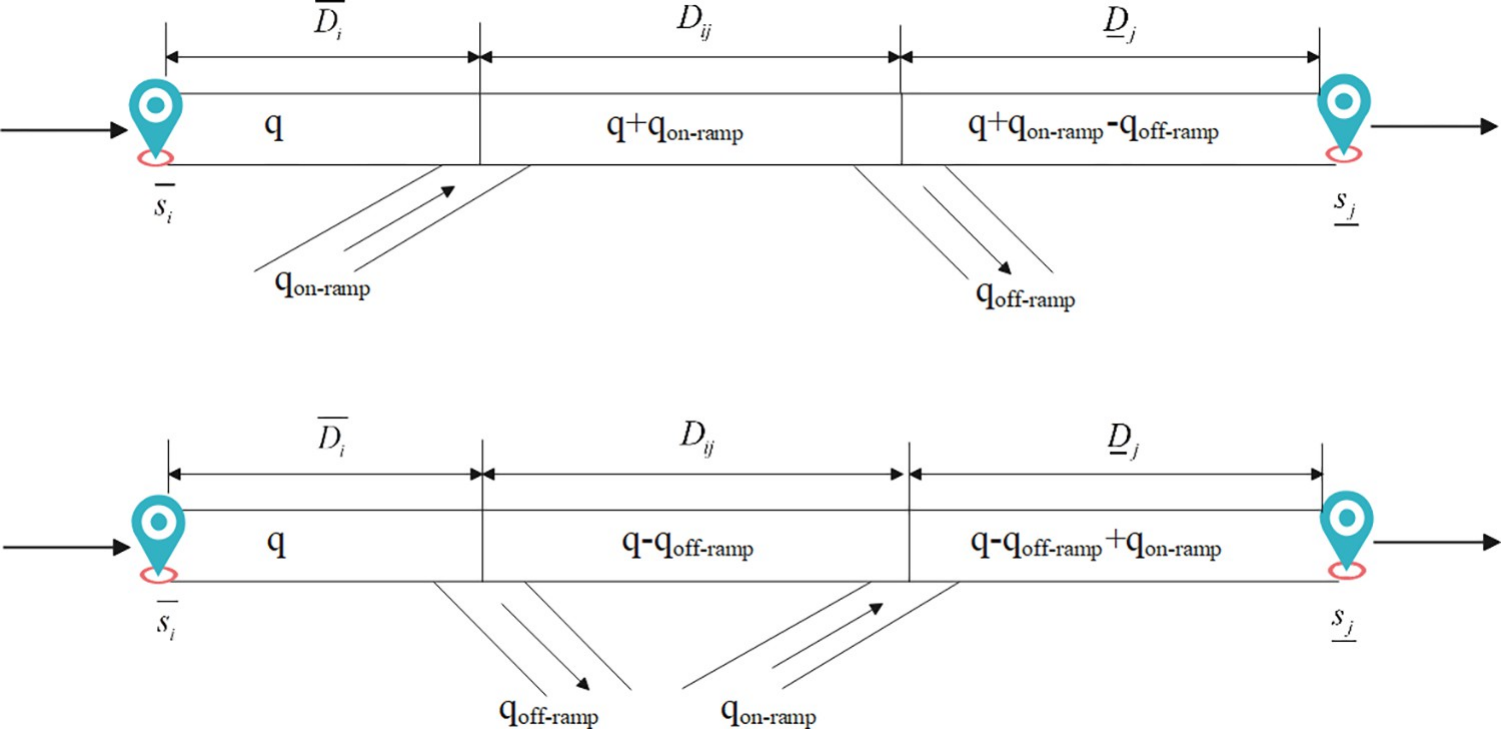
The second type of ramp-weave section.

The second type incorporates adjacent weaving segments, which are displayed in Fig 2. Since node *i* and node *j* are in close proximity to each other, the emergency vehicle may be affected by merging traffic flow *q*+*q*_*on−ramp*_ when traveling on road section *D*_*ij*_, and be affected by diverging traffic flow *q*+*q*_*on−ramp*_−*q*_*off−ramp*_ when traveling on road section *D*_*j*_. Therefore, the velocity of emergency vehicles may vary twice. The travel time at this node *Q*_*ij*_(*t*) is:

$$\theta_{ij}(t)=\frac{\bar{D_{i}}+D_{ij}/2}{v_{\bar{s_{i}}}(t)}+\frac{D_{ij}/2+{\underline{D}}_{j}}{v_{\underline{s_{j}}}(t)}$$ (23)

## Modified cuckoo search algorithm

Our MIPSSTW model integrates a VRP problem with the time consumption of nodes and arcs considering actual traffic conditions, which is an NP-hard optimization problem. In practice, commercial solvers and exact solution methods struggle to solve large instances. Some heuristic algorithms may become trapped in local optima leading to low operational efficiency and weak robustness. Thus, we introduce the cuckoo search (CS) intelligent algorithm to solve this combinational problem in this paper.

### 4.1 Standard CS algorithm

**Fig 3:**
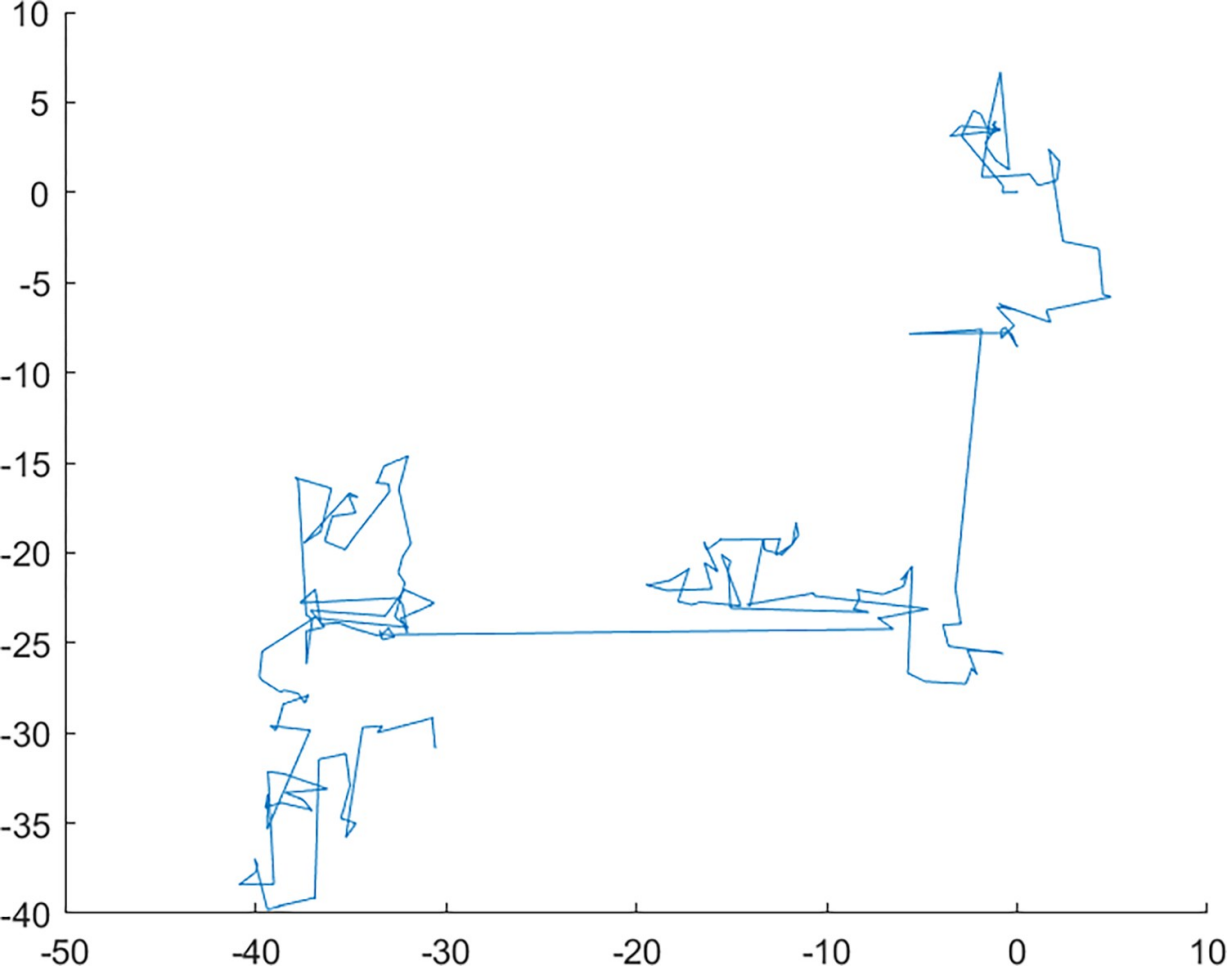
The trajectory of Lévy flight.

The generic framework of the CS algorithm draws inspiration from the interesting feature of parasitism of cuckoo species and was initially proposed by Yang and Deb in 2009 [23]. Cuckoos exhibit brood parasitism and lack the behavior of hatching eggs by themselves. Thus, they need to search for high-quality nests and foster parents for incubation and chick-rearing [35]. Cuckoo birds have evolved a remarkable ability to lay eggs that closely resemble those of the host species in color, pattern, size, and shape. Their search process is implemented through Lévy flight. Lévy flight simulates the stochastic movement of insects and birds in mathematical modeling. As shown in Fig 3, the trajectory of Lévy flight represents the random movement of insects and birds, encompassing both short-distance turns and long-distance jumps. This characteristic enables the CS algorithm to undertake large-scale exploration and fine-tuned adjustments to find the optimal fitness value. Lévy flight is modeled as a probability density function with a power-law tail. According to the Central Limit Theorem, Lévy flight tends to reach a stable distribution after a sufficient number of random walks. The new solution $X_{i}^{t+1}$ for cuckoo *i* by Lévy flight is as follows:

$$X_{i}^{t+1}=X_{i}^{t}+\alpha ⊕Levy(s,\lambda )$$ (24)

Where step size *α*(*α*>0) is decided by the scale of the related research problem, and *α* = 1 is commonly applied in many cases. ⊕ denotes central multiplicative. Similar to the Particle Swarm Optimization (PSO) algorithm, the Central Limit Theorem is more effective in the CS algorithm, as Lévy flight exhibits longer step lengths in long distances. *Levy*(*s*,*λ*) denotes the random search path and its performance depends on the step size *s* and length randomly drawn from the Lévy distribution:

$$Le^{\prime }vy(s,\lambda )=s^{-\lambda }\;1<\lambda \leq 3$$ (25)

Where the parameter *λ* denotes the average value or expected value of events within a unit of time. In summary, Lévy flight focuses on searching around solutions and occasionally taking longer steps to minimize the probability of getting stuck in local optima.

CS algorithm is governed by three ideal rules: (1) Every cuckoo lays only one egg in a random nest where one egg represents one solution. (2) At the end of each iteration, the best nest with high-quality eggs is preserved for the next generation. (3) The number of host nests is fixed. Suppose the probability of discovering the cuckoo egg for a host bird is *P*_*α*_∈(0,1). If the host bird finds a cuckoo egg, it will throw away this egg or abandon the nest. Hence, the egg will be hatched again, that is, this solution will be eliminated in the CS algorithm.

Compared to other heuristic algorithms, the CS algorithm could strike a good balance between local search strategies and overall space utilization, which has demonstrated excellent performance in terms of convergence speed and implementation. The CS algorithm has been proven successfully to solve a wide variety of multi-objective optimization problems. However, the standard CS algorithm has its limitations. It heavily relies on the random step size of Lévy flights during the searching and optimization process. The lack of communication with superior individuals leads to slow convergence and low precision in later iterations. Therefore, in order to improve the standard CS algorithm, researchers have paid more attention to exploring the variants of the CS algorithm for more complicated cases. In this paper, we develop an MCS algorithm to enhance the effectiveness of the standard CS algorithm via tuning parameters and diversity strategies.

### 4.2 MCS algorithm

This proposed variant of the CS algorithm adopts an effective multiple neighborhood structure and a path relink strategy to strengthen the exploration capability and facilitate escaping from local optima. Previous studies [36,37] have demonstrated that the neighborhood structures of 2-opt, 3-opt, OR-opt, and double bridge play a crucial role in providing benefits for a more effective random sequence in the search space. In addition, path relinking strategies (PRS) of Lévy flights are introduced to improve the diversity of exploration between initial solutions. The dynamic inertia weight strategy for the probability of eggs being discovered is designed to balance the performances of local search and global search. The implications of these key elements and improvements of the MCS algorithm are detailed below.

#### Nest

A nest serves as a container for an individual within the population, and the number of nests is fixed. Given that a cuckoo usually lays only one egg in the nest, the quantity of nests involved in this combination problem corresponds to the size of the population in the MCS algorithm.

#### Egg

An egg represents a solution laid by a cuckoo in the nest. In our study, one egg is encoded as a response route for emergency vehicles.

#### 4.2.3 Dynamic inertia weight strategy for portion of bad solutions *P*_*a*_

In nature, if a host bird detects alien eggs, it will throw out this egg or abandon the nest, preventing the cuckoo’s eggs from hatching. In the CS algorithm, the portion of throwing alien eggs or abandoning its nest is regarded as a bad solution and will be promptly replaced by a superior alternative. We assume that the probability of the host bird abandoning eggs is denoted as *P*_*a*_. In practice, this probability *P*_*a*_ plays an essential role in determining the final fitness value, for it affects the decisions and behaviors at different stages of the simulation. Its dynamics are shaped by factors such as environmental conditions, the behavior strategy of cuckoo birds, the behavior of host birds, and so on. These factors may lead to corresponding variations in the interactions between cuckoo and host birds. For instance, when the fraction of worse solution *P*_*a*_ is closer to 1, it illustrates a higher probability of discovering the alien eggs. A low number of nests for cuckoo egg diminishes the diversity and effectiveness of the CS algorithm. Oppositely, if the portion *P*_*a*_ is minute and close to zero, the performance of the CS algorithm is specialized in the global search, but the ability of local search is weak. On the other hand, the portion of bad solutions *P*_*a*_ of the basic CS algorithm is arbitrarily set as a constant resulting in the randomness of the precision of final solutions. In extreme cases, this randomness might lead to the abandonment of the high-quality eggs located at better nests, which misses the optimal solution. Therefore, we explore a dynamic inertia weight strategy for the fraction of worse nests *P*_*a*_ to enhance the selection capability of the CS algorithm. The specific expression is:

$$newP_{a}=\omega \cdot P_{a}$$ (26)

$$\omega =\omega_{\max}(\omega_{\max}-\omega_{\min})\cdot r-\varphi \cdot frnd()$$ (27)

Where *ω* denotes the inertial weight coefficient and depends on its maximum value *ω*_max_ and minimum value *ω*_min_. The coefficient *r* is a random value that follows a (0,1) uniform distribution and aims to allow the inertial weight coefficient *ω* could randomly take any value within the range [*ω*_max_−*ω*_min_] in order to reinforce the flexibility of the MCS algorithm. The coefficient *φ* and function *frnd*() are designed for the deviation of the inertial weight *ϕ*. Function *frnd*() generates a stochastic value conforming to an asymmetric F-distribution. The asymmetric F-distribution is a variant of the F-distribution characterized by the tendency, non-uniformity, and asymmetry. These characteristics have a significant impact on both the uniformity of the solution set and the augmentation of diversity.

As shown in Formula (27), the inertial weight coefficient *ω* undergoes size fluctuations during the whole iteration. When the inertial weight coefficient *ω* is small, the MCS algorithm enhances the diversity of the solution space and is able to avoid trapping in local optima. Additionally, the probability of abandoning the worst solution *P*_*a*_ intensifies in tandem with the growth of the inertial weight coefficient *ω*. This will reduce the number of solutions in the space and improve to converge quickly.

#### 4.2.4 Path optimization of Lévy flight

Lévy fight plays a vital role in modeling the navigational patterns of cuckoo birds during searching for the nests of other host birds. As shown in Formula (25), Lévy flight refers to both step size and direction for establishing new nests. In the standard CS algorithm, the long jumps of Lévy flight have the potential to skip over regions within the search space incorporating better solutions, which leads to premature convergence towards suboptimal solutions. Moreover, Lévy flight in the basic CS algorithm performs ineffectively in specific terrain types, particularly those characterized by narrow, deep valleys. It may have difficulties in local search around identified regions of interest. In order to address these limitations, a multi-neighborhood structure and path relinking strategies(PRS) are used to improve the diversity of exploration routes and facilitate the selection of the optimal route.

A multi-neighborhood structure consists of four intra-routes, as shown in Table 2. These intra-routes, namely 2-opt, 3-opt, OR-opt, and double-bridge moves, involve changes occurring within the same route. Each neighborhood structure is associated with specific step sizes of Lévy flight and generates a new solution by rearranging the sequence of visited nodes and arcs within another existing solution. This strategic integration aims to overcome the shortcomings of the standard CS algorithm and improve its performance across various landscapes.

**Table 2:** Neighborhood structures [36,37].

Name	Details
**2-opt**	Two arcs that are not adjacent are removed and later a fresh initial solution will be inserted into the route.
**3-opt**	Three arcs that are not adjacent are removed and later a fresh initial solution will be inserted into the route.
**OR-opt**	Two adjacent arcs are removed and later a fresh initial solution will be inserted into the route.
**Double bridge**	Four arcs are removed and incorporated into a route. They don’t necessarily have to be in consecutive order to generate a novel initial solution."

In order to enhance the survival chances of cuckoo bird eggs, these avian species are compelled to refine their evolution skills. The PRS for Lévy flights is adopted to optimize their trajectories between high-quality selected solutions. When the count of step sizes is below 2, the inter-route PRS involves the exchange of one node from one route with a node from another route. Conversely, if the number of step sizes exceeds 2, the cross-route PRS may engage in combining elements from one path with elements from another path to generate a new route and solutions. This strategy of path operations highlights more intensification and diversity in the search space to escape the MCS algorithm from the local optimum effectively.

### 4.3 Flowchart and pseudocode of MCS algorithm

**Fig 4:**
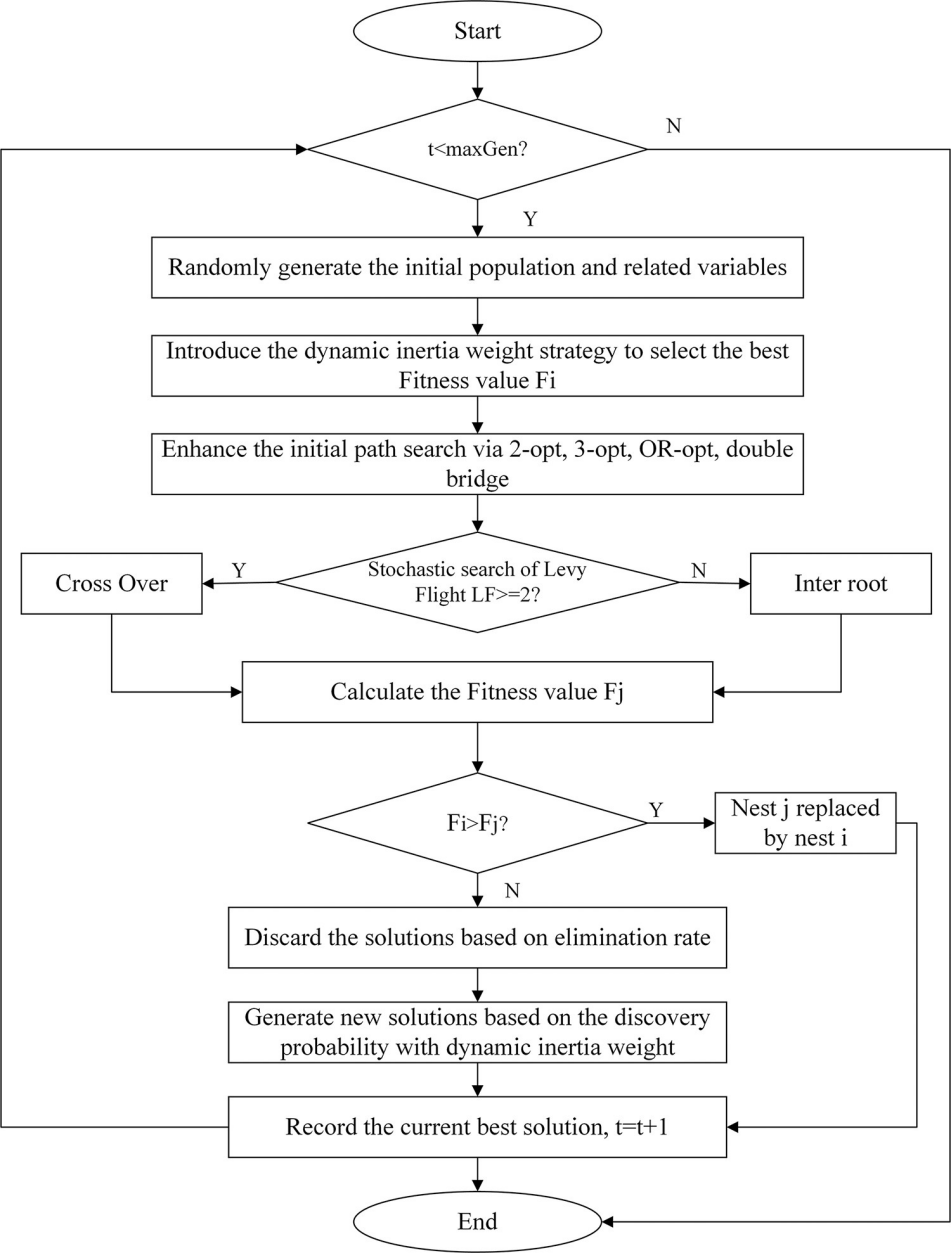
The flowchart of MCS algorithm.

**Fig 5:**
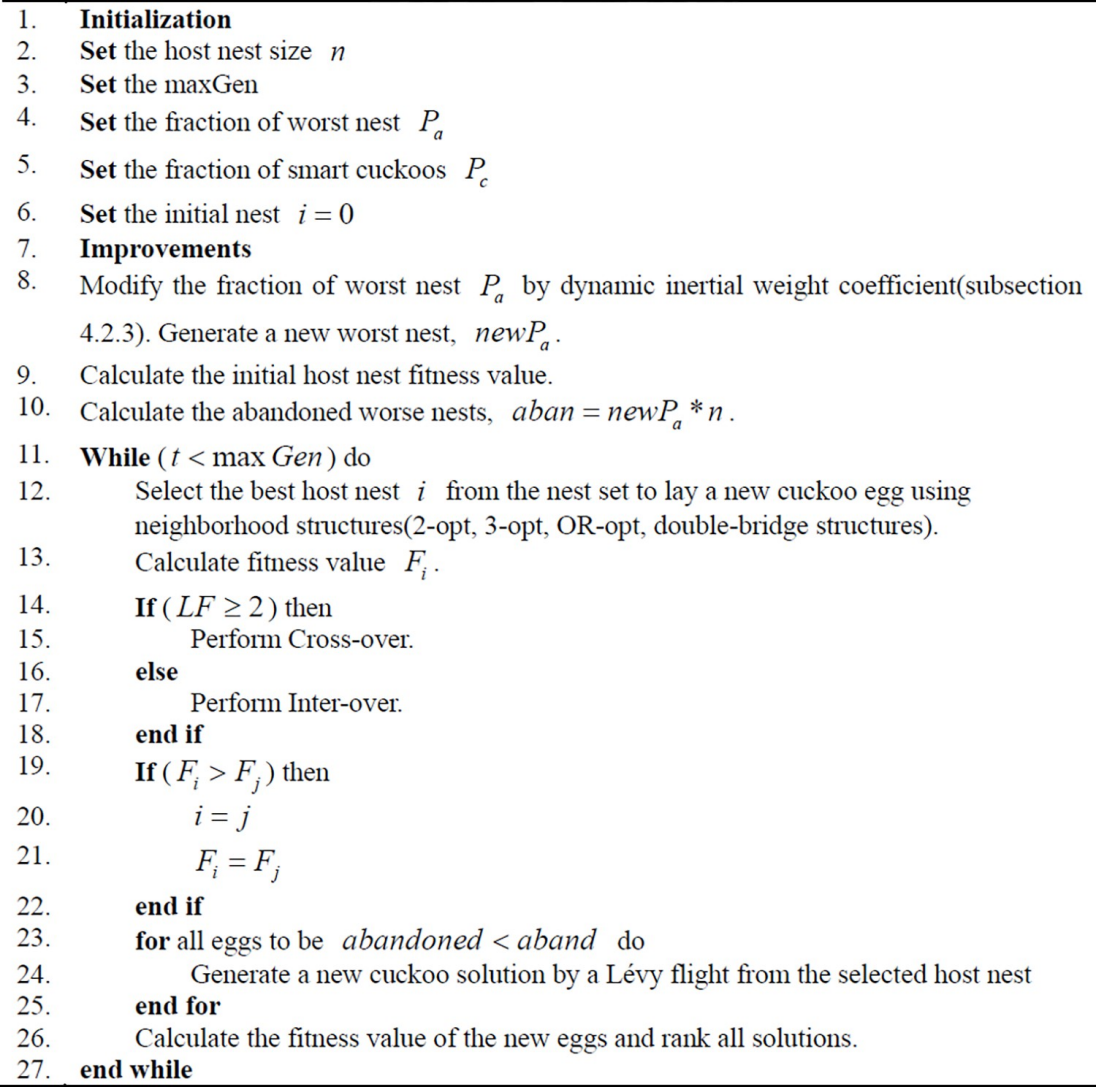
The pseudocode of the MCS algorithm.

The MCS algorithm amalgamates the strengths of the standard CS algorithm and enhances the search capability of cuckoo birds and the discovery ability of alien eggs of host birds. This improvement is achieved through the implementation of a dynamic inertia weight strategy and path optimization of Lévy flight. Fig 4 displays the flowchart of the MCS algorithm. The pseudocode of the MCS algorithm is shown in Fig 5.

## 5. Case study

### 5.1 Experiment design

**Fig 6:**
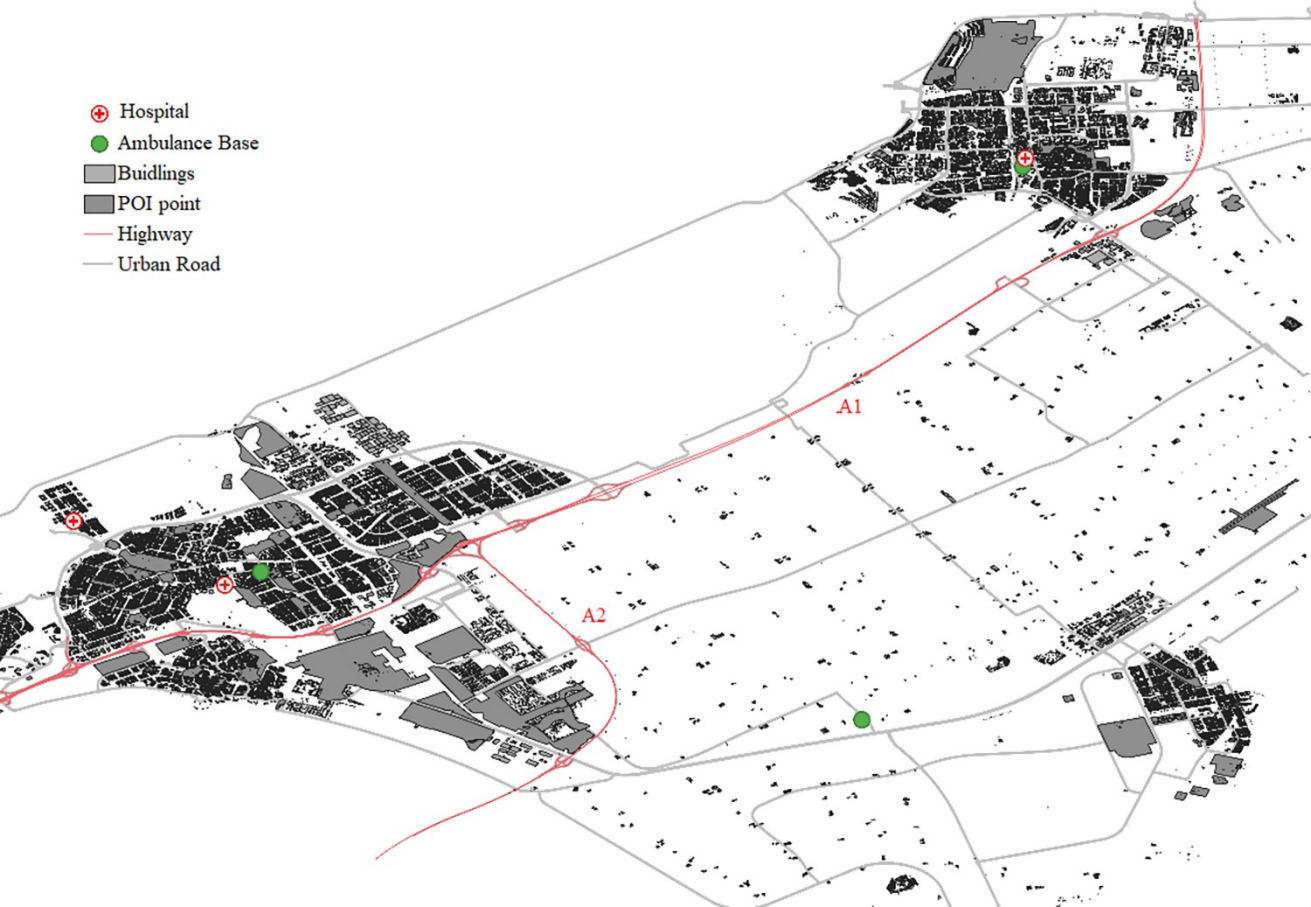
The map of the research area.

In this section, we design computational experiments to verify the efficacy and effectiveness of our MIPSSTW model and MCS algorithm in optimizing EMS ambulance routing. The research area contains three hospitals and three ambulance bases, as shown in Fig 6. It is noteworthy that the hospital and ambulance base in the northeast direction are considered the same location due to their close proximity. Excluding this particular node, the remaining ambulance bases and hospitals are situated in distinct locations. In addition, the research area is served by two intersecting highways, namely A1 and A2, both delineated by red lines on the map. The detailed experimental setup aims to provide a comprehensive assessment of the proposed MIPSSTW model and MCS algorithm in optimizing ambulance routing for EMS in a real-world scenario within the specified region in China.

**Fig 7:**
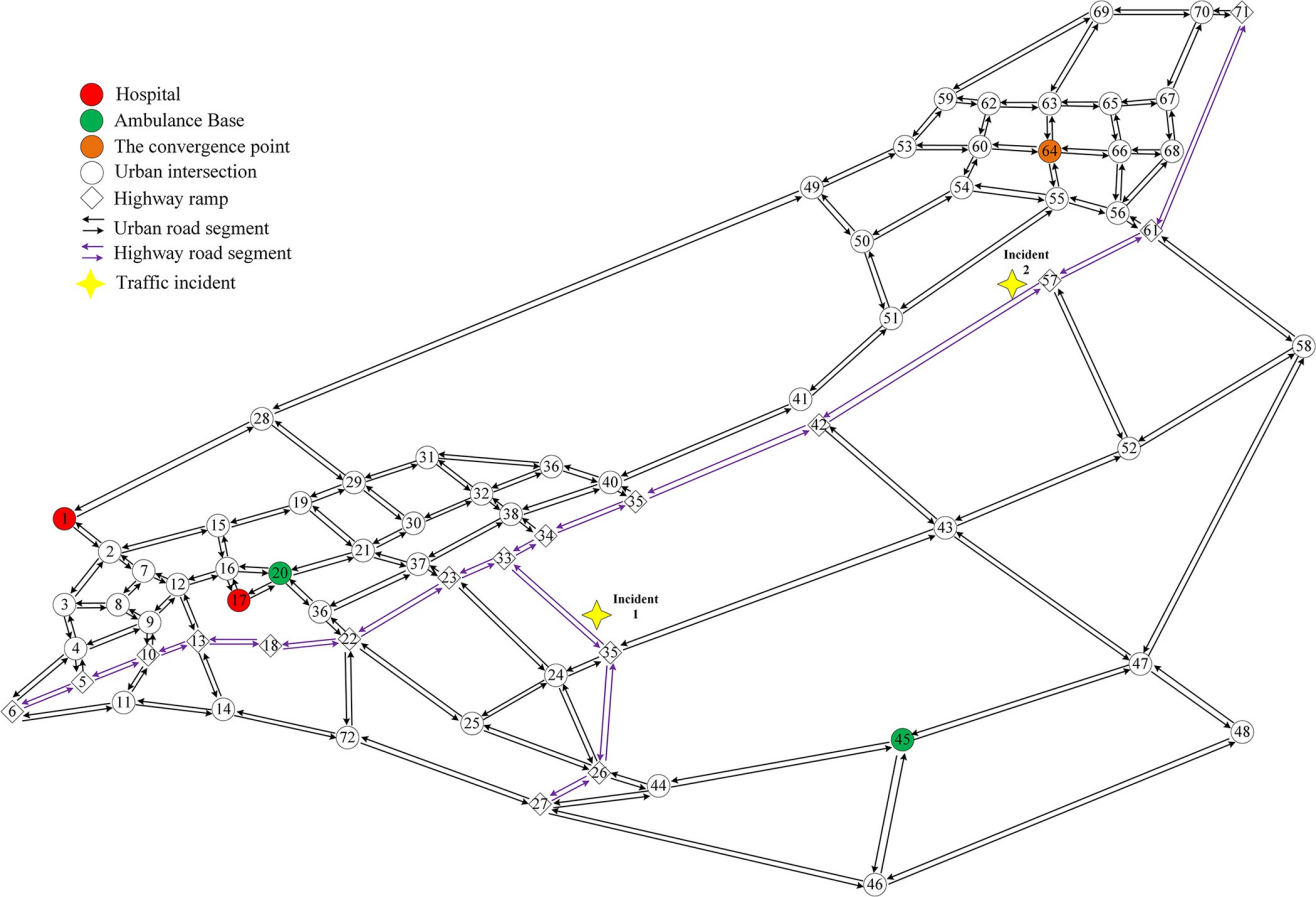
The topological diagram of the road network.

Based on the actual road structure shown in Fig 6, we have undertaken simplifications to represent micro roads, residential roads, and some impassable roads in the specified area. The outcome of the topology road network is illustrated in Fig 7. The framework of this topology road network consists of a total of 71 nodes including intersections, highway ramps, hospitals, and ambulance bases. There are 123 road segments incorporating 109 urban road sections with various road grades and 14 highway segments. Nodes 20 and 45 serve as ambulance dispatch bases, and nodes 1 and 17 represent hospital locations. As mentioned above, the convergence node, denoting the shared location of the hospital and ambulance dispatch base, is marked as node 1. The refined representation of the road network provides a clear and simplified view, which enables a more focused analysis within the context of the EMS ambulance routing optimization.

We select two real-life incident scenarios to structure our experiments and validate the effectiveness of the MIPSSTW model. Based on actual traffic accident data, incidents on the highway network are annotated as nodes 35 and 57. Our goal is to assess the necessity of considering complex traffic conditions for optimizing emergency vehicle routing. Therefore, we compare ambulance routing optimization approaches with and without the consideration of time consumption for nodes and arcs in the experiments. Furthermore, the proposed MCS algorithm is coded in MATLAB 2020 using an Intel Core i7 processor, PCs with 3.07GHz and 8GB RAM memory. The performance of the MCS algorithm is evaluated through comparisons with the standard CS algorithm and other classic heuristic algorithms. Parameters for the MCS algorithm are set following the guidelines provided in literature [25], as detailed in Table 3.

**Table 3:** Parameters setting in the MCS algorithm.

Parameters	Values
Initial population	100 set in the preliminary experiment
MaxGen	40
Portion of bad solutions *P*_*a*_	0.25
New portion of bad solutions *newP*_*a*_	[0.2,0.5]
Portion of smart cuckoo *P*_*c*_	0.6

### 5.2 Result and discussion

**Fig 8:**
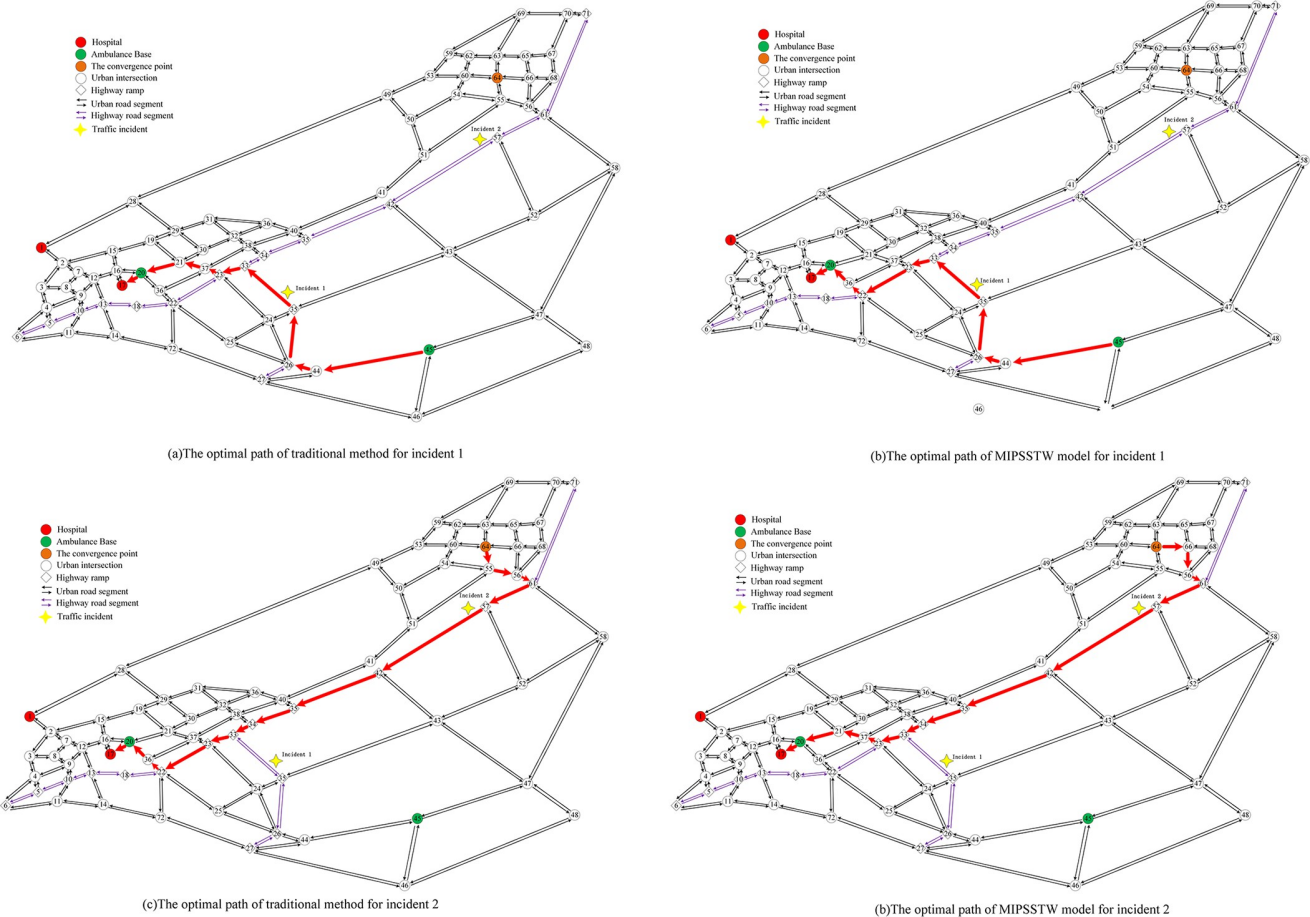
The optimal paths of different methods for incident 1 and incident 2.

The results of the comparison between our MIPSSTW model and the approaches that neglect the time consumption of nodes and arcs are summarized in Table 4. We have considered four classic estimated indicators: travel time, average speed, distance, and average delay. Fig 8 shows the schematic diagram of emergency routing for both the traditional method and our model. The traditional method operates under the assumption that ambulances face no obstruction from other vehicles on the road network, that is, overlooking complex traffic conditions. However, our model takes the factors of time consumption of intersection, ramps, and other impedance for the highway incident into account. For accident 1, originating from node 45 and destined for node 17, the traditional approach yields an optimal path of 45-44-26-35-33-23-22-36-20-17. In contrast, our proposed model considering the traffic conditions, suggests a more efficient route of 45-44-26-35-33-23-37-21-20-17. Despite a 1.5km increase in distance, the travel time and average delay decreased by 3.7 mins and 3.2 mins, respectively. The average speed also experiences an improvement of 8.8km/h. The optimization ratios are 12%, 31%, and 21%, respectively. As for accident 2, where the ambulance originates from node 64 with the designated hospital at node 17., our model achieves a reduction of 2.2 mins in travel time and an enhancement of 7.9 km/h in average speed compared to the traditional method. The average delay is notably decreased by 28%. These findings highlight the superior performance of our MIPSSTW model in optimizing emergency routing by accounting for real-time traffic conditions and various impedances, which represents significant improvements in key performance indicators.

**Table 4:** The comparison of routing optimization approaches.

Incident No.	Rescue routing and performance metrics	Traditional approach	MIPSSTW model
**Incident 1**	**Rescue routing**	45-44-26-35-33-23-22-36-20-17	45-44-26-35-33-23-37-21-20-17
	**Travel time(min)**	31.2	27.5
	**Average speed(km/h)**	40.8	49.5
	**Distance(km)**	21.2	22.7
	**Average delay(min)**	10.4	7.2
**Incident 2**	**Rescue routing**	64-55-56-61-57-42-35-34-33-23-22-36-20-17	64-66-56-61-57-42-35-34-33-23-37-21-20-17
	**Travel time(min)**	33.5	31.3
	**Average speed(km/h)**	50.9	57.9
	**Distance(km)**	28.4	30.2
	**Average delay(min)**	8.5	6.1

Table 4 unmistakably demonstrates the superiority of routing optimization that takes into account complex traffic conditions compared to traditional approaches. Traditional path search processes typically provide a rough evaluation of the travel time of emergency vehicles based on the traffic impedance of road segments, offering a limited reflection of actual traffic conditions. However, most of the stop- and go- conditions occurring at signalized intersections and ramp-weave sections, are not accurately captured by the traditional method. Especially when the traffic flow reaches or exceeds the capacity of the road section, the phenomenon of traffic flow breaking down incurs ambulance speed drops. The traditional method falls short in ensuring the optimal routing of emergency vehicles in the absence of considerations for complex traffic conditions. in contrast, our model comprehensively analyzes the characteristics of crucial compositions of the emergency vehicle path. We respectively apply an improved BPR function for road segments, a prediction method based on queueing theory for intersections on the urban road network, and an improved fixed detector method for ramp-weave sections on the highway network. Factors like signal light changes, queue lengths, and the speed of merging and diverging at ramps may lead to the randomness of the selection of rescue routing. The MIPSSTW model proves to be more effective in anticipating these challenges, consequently ensuring optimal routing with the shortest response time. This improvement is expected to significantly enhance the efficiency of emergency management for highway incidents.

**Fig 9:**
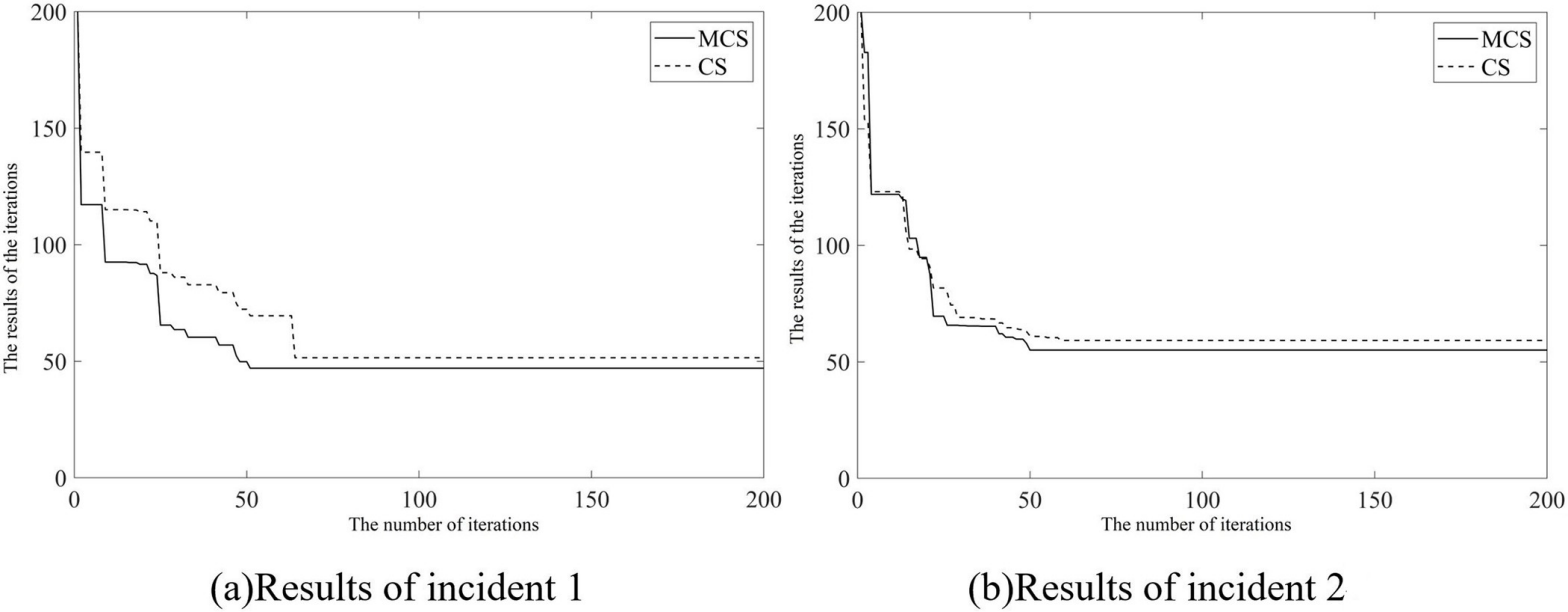
The comparative results between CS and MCS algorithms.

In addition, we investigate the performance of dynamic inertia weight strategy and path optimization of Lévy fight on a randomly generated solution to validate the effectiveness of the MCS algorithm in the VRP. Several comparative experiments involving different solving algorithms were designed for this purpose. For the response routing for incidents 1 and 2, we explore the performance of both the standard CS algorithm and MCS algorithm within the context of our MIPSSTW model. The results of the CS algorithm and MCS algorithm are illustrated in Fig 9. As shown in Fig 9, it is observed that in the optimal path-solving for incidents 1 and 2, the MCS algorithm exhibits a faster convergence speed compared to the standard CS algorithm. Especially in the iteration process of incident 2, the CS algorithm initially converges faster than the MCS algorithm. However, the MCS algorithm ultimately achieves the optimal vehicle path with fewer iterations. This observation suggests that the neighborhood structures and PRS strategies in the MCS algorithm strengthen the diversity of exploration between initial solutions. The dynamic inertia weight strategy governing the probability of discovering eggs effectively balances the performances of local search and global search. Therefore, the MCS algorithm has been demonstrated that it has a higher likelihood of searching for the optimal solution, faster convergence speed, and more robust global search capabilities, which significantly improves the CS algorithm’s running efficiency.

**Fig 10:**
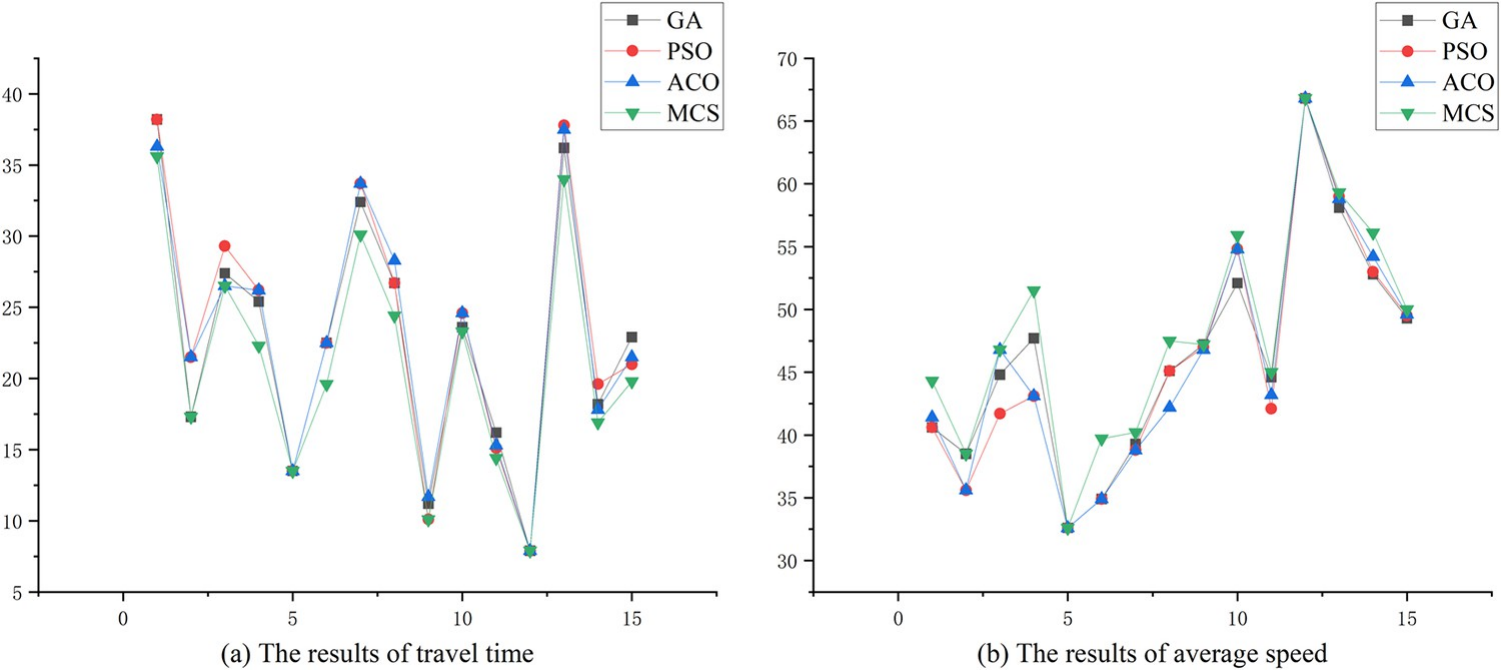
The comparative results of travel time and average speed of four algorithms.

Finally, we also conducted a comparative analysis of the performances of the MCS algorithm against three other commonly used swarm intelligence algorithms: Genetic Algorithm(GA), Particle Swarm Optimization(PSO), and Ant Colony Optimization(ACO). In order to ensure robustness, 50 different simulation experiments were randomly conducted based on historical incident data. We then selected 15 results shown in Table 5 and Fig 10. The evaluation criteria for the algorithms’ performances are the travel time and average speed associated with the optimal routing. Due to the relatively close distance and poor road conditions in incident 5, there is no significant difference in the travel time of the optimal routings among these four algorithms. However, the MCS algorithm comparatively performs well in other computational results and helps to reduce the travel time of ambulances during the rescue process. A similar situation also happens in incident 12, that is, the results represent the same trend among the four algorithms. This is because the incident occurred at night and the selected routing in the road network is limited leading to similar results at last. The ability of the MCS algorithm to consistently deliver favorable results in diverse scenarios indicates its potential as a robust optimization tool for emergency vehicle routing in various conditions.

**Table 5:** The results of four algorithms.

No.	Algorithm							
	GA		PSO		ACO		MCS	
	Travel time (min)	Average speed (km/h)	Travel time (min)	Average speed (km/h)	Travel time (min)	Average speed (km/h)	Travel time (min)	Average speed (km/h)
**1**	38.2	40.6	38.2	40.6	36.3	41.4	35.6	44.3
**2**	17.3	38.5	21.5	35.6	21.5	35.6	17.3	38.5
**3**	27.4	44.8	29.3	41.7	26.5	46.8	26.5	46.8
**4**	25.4	47.7	26.2	43.1	26.2	43.1	22.3	51.5
**5**	13.5	22.6	13.5	22.6	13.5	22.6	13.5	22.6
**6**	22.5	34.9	22.5	34.9	22.5	34.9	19.6	39.7
**7**	32.4	39.3	33.7	38.8	33.7	38.8	30.1	40.2
**8**	26.7	45.1	26.7	45.1	28.3	42.2	24.4	47.5
**9**	11.2	47.2	10.1	47.0	11.7	46.8	10.1	47.2
**10**	23.6	52.1	24.6	54.8	24.6	54.8	23.3	55.9
**11**	16.2	44.6	15.1	42.1	15.3	43.2	14.4	45.0
**12**	7.9	66.8	7.9	66.8	7.9	66.8	7.9	66.8
**13**	36.2	58.1	37.8	59.0	37.5	58.8	34.0	59.3
**14**	18.2	52.8	19.6	53.0	17.8	54.2	16.9	56.1
**15**	22.9	49.3	21.0	49.5	21.5	49.6	19.8	50.0

## 6. Conclusions

This research work discussed a MIPSSTW-based optimization model for EMS ambulances considering complex traffic conditions for highway incidents. The purpose of this study is to bring insight into emergency vehicle routing problems, contributing to the enhancement of the effectiveness of the prehospital healthcare system from both theoretical and practical perspectives. In this study, we specifically analyze the evaluation approaches for vital compositions of the road network. The estimation approach for travel time incorporates an improved BPR model for road segments, the prediction method based on queueing theory for intersections on the urban road network, and an enhanced fixed detector method for ramp-weave sections on the highway network. In addition, we have extended and improved the standard CS algorithm via the dynamic inertia weight strategy and neighbor structure-based path optimization of Lévy flight. The strategy of inertial weight for the MCS algorithm dynamically tunes the fraction of worse nests. It compensates for the potential shortcoming of slow convergence and low search accuracy in the later stage of the standard CS algorithm. The multiple neighborhood structures and path relinking strategies are beneficial to obtain the best solution, striking a higher probability of balancing the local search and global search. The computational results based on Chinese EMS data have verified the significance of our MIPSSTW model and MCS algorithm in improving the efficiency of emergency vehicle rescue. Our contributions serve as valuable tools for real-world EMS decision-making, adaptable to various emergency scenarios. These findings offer actionable insights for practical applications and contribute to the broader field of emergency management.

In the future, the research work can be extended to explore the patient transportation problem considering transport emissions. Analyzing traffic emissions under saturated and oversaturated traffic conditions plays a vital role in environmental sustainability and public health. It is a challenging task to study energy conservation and emissions reduction within the context of emergency management. In addition, our MCS algorithm concentrates on the improvement of the diversity of initial solutions and search mechanisms. It may confront the challenges of inadequate search capabilities and low accuracy in obtaining non-dominated solutions when applied to high-dimensional multi-objective optimization problems. Therefore, our further work will modify the MCS algorithm to solve high-dimensional multi-objective optimization problems with multiple depots for more practical applications.

## Supporting information

S1 File Part of the accident data. (XLSX)
